# GLI1 Inhibitor SRI-38832 Attenuates Chemotherapeutic Resistance by Downregulating NBS1 Transcription in BRAF^V600E^ Colorectal Cancer

**DOI:** 10.3389/fonc.2020.00241

**Published:** 2020-02-28

**Authors:** Ruowen Zhang, Jinlu Ma, Justin T. Avery, Vijaya Sambandam, Theresa H. Nguyen, Bo Xu, Mark J. Suto, Rebecca J. Boohaker

**Affiliations:** ^1^Southern Research, Division of Drug Discovery, Birmingham, AL, United States; ^2^Department of Radiation Oncology, First Affiliated Hospital, Xian Jiaotong University, Xi'an, China; ^3^Tianjin Medical University Cancer Institute and Hospital, Tianjin, China

**Keywords:** colorectal cancer, 5-FU resistance, DNA damage repair, GLI1 target gene, novel GLI1 inhibitor, target therapy

## Abstract

Resistance to radiation and chemotherapy in colorectal cancer (CRC) patients contribute significantly to refractory disease and disease progression. Herein, we provide mechanistic rationale for acquired or inherent chemotherapeutic resistance to the anti-tumor effects of 5-fluorouracil (5-FU) that is linked to oncogenic GLI1 transcription activity and NBS1 overexpression. Patients with high levels of GLI1 also expressed high levels of NBS1. Non-canonical activation of GLI1 is driven through oncogenic pathways in CRC, like the BRAF^V600E^ mutation. GLI1 was identified as a novel regulator of NBS1 and discovered that by knocking down GLI1 levels *in vitro*, diminished NBS1 expression, increased DNA damage/apoptosis, and re-sensitization of 5-FU resistant cancer to treatment was observed. Furthermore, a novel GLI1 inhibitor, SRI-38832, which exhibited pharmacokinetic properties suitable for *in vivo* testing, was identified. GLI1 inhibition in a murine BRAF^V600E^ variant xenograft model of CRC resulted in the same down-regulation of NBS1 observed *in vitro* as well as significant reduction of tumor growth/burden. GLI1 inhibition could therefore be a therapeutic option for 5-FU resistant and BRAF^V600E^ variant CRC patients.

## Introduction

Colorectal Cancer (CRC) is ranked as the third most prevalent and deadly tumor type in the United States, with ~145,000 newly diagnosed cases and upwards of 50,000 deaths attributed to the disease in 2018 alone (1). A predominant driver of this mortality rate is inherent or acquired resistance to the standard-of-care combination regimens with the anti-metabolite 5-Flurouracil (5-FU) as the base therapy (i.e., FOLFOX and FOLFIRI). Genetic analysis of biopsied tissue from CRC patients indicates that a significant subset, ~10%, expressed oncogenic BRAF (2, 3). Within this subset, patients bearing a missense mutation within BRAF at position V600E faced significantly shorter overall survival and an 80% mortality rate (4).

Attempts to specifically target this *BRAF*^*V*600*E*^ variant with the single-agent kinase inhibitor Vemurafenib was found to be wholly ineffective with a <5% response rate, leaving only very aggressive and cytotoxic combinations as treatment options (5). Despite the inefficacy of targeted therapies and the aforementioned resistance to standard-of-care, the strategy remains centered on 5-FU, ionizing radiation therapy, and other DNA damaging agents (6, 7). This strategy relies on the induction of an overwhelming and cytotoxic threshold of double-stranded breaks (DSBs) in the DNA, chromosomal instability, and subsequent activation of the apoptotic cascade (8–11). Resistance to DNA damaging agents in this subset of patients can be attributed to a *BRAF*^*V*600*E*^ driven hyperactive DNA damage repair (DDR) process that outpaces the induction of genotoxic stress and accumulation of DNA damage (12).

The DDR machinery is comprised of multiple sensors and repair enzymes that are deployed at various stages of the cell cycle to ensure the maintenance of chromosomal integrity and replicative fidelity. Numerous reports of overexpression of critical DDR component proteins in oncogenic environments indicate that chemo-resistance can arise due to over-activation of the MRE11, Rad50, NBS1 (MRN) complex. A critical component of the MRN complex is the Nijmegen breakage syndrome-1 (NBS1; p95, nibrin) protein, produced by *NBS* gene. Complexing with MRE11 and RAD50, NBS1 is the first factor to detect and bind to histone H2AX at the site of a DNA lesion which subsequently forms the multimeric MRN complex, initiating the process of DSBs repair (13–16). Overexpression of individual components of the MRN complex has been significantly associated with adverse clinical outcomes of including poor prognosis relative to insufficient chemotherapeutic response (17). For example, at a cellular level, down-regulation of NBS1 by transfecting domain-negative NBS1 significantly increased chemo-sensitivity to cisplatin *in vivo* (18). This indicates that total protein level, rather than post-translational modification is critical in the correlation of hyperactive DDR and poor prognosis.

In an effort to identify drivers of MRN-initiated repair activity, we examined the role of oncogenic transcription elements that could contribute to overexpression, and subsequent hyper-activation, of DSB repair mechanisms. Of particular interest are GLI family proteins, transcriptional effectors of the Hedgehog (Hh) pathway that are typically active in normal development. GLI1 and GLI2 serve as transcriptional promoters, conversely GLI3 acts as a transcriptional repressor. Of the three factors, GLI1 has emerged as a critical oncogene, having transcriptional activity as the distal effector of both the canonical Hh pathway as well as the non-canonical oncogenic KRAS/BRAF pathway (19). Additionally, oncogenic GLI1 activity is often linked to chemo-resistance (20–25) due in part to GLI1's transcriptional regulation of several oncogenes involved in critical processes at all stages of cellular life ranging from proliferation and survival to cycle checkpoints (26). We have previously reported that GLI inhibition led to significant DNA damage, and subsequent apoptosis in colon carcinoma cells due to an extended pause in DNA replication licensing via down-regulation of CDT1, a critical DNA replication licensing factor and transcriptional target of GLI1 (27). However, transient overexpression of CDT1 only partially reduced the cleavage of caspase-3 and merely rescued GLI1 inhibition-induced cytotoxicity, while γH2AX, a DNA damage marker, still consecutively accumulated. Based on this phenomenon, we hypothesized that inhibition of GLI1 may not only induce DNA damage, but also impede DNA damage repair process via coincidental transcriptional inhibition of DDR component genes. Indeed, examination of the promotor regions of MRN component proteins indicate that NBS1 is a putative target for GLI1 transcription.

Herein, this study illustrates the clinical relevance of GLI1 and NBS1 overexpression to poor clinical outcome in CRC patients treated with 5-FU based therapeutics. A series of biochemical and genetic assays elucidates a mechanism by which *BRAF*^*V*600*E*^ mutant cancers circumvent the efficacy of 5-FU induced DNA damage through a previously undescribed GLI1-mediated transcription of the DNA damage-sensing protein NBS1. Additionally, we demonstrate that the zinc-finger domain of GLI1 can be targeted to prevent binding to the conserved consensus sequence within the *NBS* promotor region, abrogating any inherent or induced resistance to 5-FU. Finally, we demonstrate proof-of-concept anti-tumor efficacy in a demonstrably 5-FU-resistant CRC xenograft model with a first-in-class direct GLI1 inhibitor, SRI-38832. Not only has the identification of SRI-38832 provided an *in vivo* tool to study GLI oncogenic activity, but also has emerged as a structural lead for further optimization.

## Materials and Methods

### Cell Culture and Reagents

The human colon carcinoma cell lines HT29, SW480, HCT8, and HCT116 cells were purchased from American Type Culture Collection (ATCC) and routinely verified by morphology and growth characteristics. Cells were cultured in 10% FBS-supplemented RPMI medium with L-glutamine and maintained at 37°C with 5% CO_2_. For Western blot analysis, antibodies against GLI1, NBS1, MRE11, RAD50, γ-H2AX, cleaved caspase-3, and β-Actin were purchased from Cell Signaling. For confocal microscopy, anti-γ-H2AX antibody was obtained from Millipore; anti-NBS1 mouse monoclonal antibody and anti-MRE11 antibody were purchased from Novus; and AlexaFluor 488 goat anti-rabbit and AlexaFluor 633 goat anti-mouse secondary antibodies were obtained from Invitrogen. GANT61 and 5-FU were purchased from Sigma. SRI-38832 was synthesized in-house at Southern Research (Birmingham, AL).

### Clinical Samples and Immunohistochemistry

The biopsies of 185 colon/rectum cancer patients were collected from the Department of Pathology at the First Hospital of Xi'an Jiaotong University (Xi'an, China) between 2014 and 2017. None of the patients received preoperative radiotherapy or chemotherapy. After de-paraffinisation and rehydration, the TMA sections were subjected to high pressure for 2 min to achieve antigenic retrieval. The slides were incubated overnight at 4°C with the following primary antibodies: NBS1 (Abcam, Cat # ab32074, 1:250) and GLI1 (Abcam, Cat # ab151796, 1:200). The sections were then incubated with DAB for 2 min. The staining results were evaluated independently by two genitourinary pathologists to determine the average percentage of stained cells.

### Western Blot Analysis

Cells were seeded in six-well plates and cultured until 70–80% confluent in 10% FBS-supplemented RPMI medium with L-glutamine and maintained at 37°C with 5% CO_2_. The cells were then treated with/without GANT61, SRI-38832, or 5-FU as indicated in the figure legends, and protein isolates were analyzed by Western blot. Briefly, 15 μg of protein was loaded and separated on a SDS-PAGE gel. Proteins were transferred to polyvinylidene difluoride (PVDF) membranes overnight. Membranes were washed in TBS-T, blotted in primary antibody overnight at 4°C, and subsequently washed and blotted with HRP-linked secondary antibody for 1 h. Membranes were developed and images were taken on an ImageQuant LAS 4000.

### Confocal Microscopy

Cells were plated at a density of 50,000 cells/well in six-well plates on coverslips and allowed to attach overnight. Media was removed and cells were treated with/without GANT61 (20 μM) for 48 h. Coverslips were removed and placed in a humidity chamber for fixation by absolute methanol for 10 min at 4°C. Cells were permeabilized with acetone for 1 min at 4°C. After washing with PBS × 3, cells were incubated with the diluted primary antibody overnight at 4°C. Cells were subsequently washed x 3 and incubated with appropriate secondary antibody at room temperature in the dark for 1 h. After washing with PBS, cell nuclei were stained with DAPI at RT for 5–10 min. Confocal images were acquired on a Nikon A1 laser confocal system with a Nikon Eclipse Ti microscope and a 60X Plan Apo objective. Lasers used were 405 nm for blue, 488 nm for green, and 561 nm for red. NIS Elements AR 4.5000 software was used to acquire Z-stacks of each channel sequentially to avoid spectral cross talk. Each slice was captured at a 0.15 μm step. Primary antibodies used were γH2AX (Millipore 1:200), NBS1 (Novus 1:200), and MRE11 (Cell Signaling 1:200). The co-localization index for specific foci and fluorescent intensity were calculated by ImageJ.

### NBS1 mRNA Extraction and Quantitative Realtime PCR (qRT-PCR) Assay

Cells were cultured and treated with/without GANT61 or SRI-38832 as indicated in the figure legends. Total RNA was extracted by Invitrogen™ PureLink™ RNA Mini Kit (Fisher scientific) and cDNAs were synthesized with the ThermoScript reverse transcriptional system. qRT-PCR was performed to assess the enrichment of the specific proteins along the NBS1 ORF using specific primer pairs: NBS1-primer-1-F:AGCAGACCAACTCCATCAGA,NBS1-primer-1-R: CAGGCTCATTCTCAGATAGA. Human GAPDH cDNA was used as an internal control.

### ChIP Analysis

HT29 cells (2 × 10^6^) were seeded in T75 flasks. After overnight attachment, cells were treated with/without GANT61 (20 μM) or SRI-38832 (20 μM) for 24 h. The cells were trypsinized, washed with 1x PBS and fixed with 1.1% formaldehyde for 10 min at RT. Glycine (0.125 M) was added to stop the reaction. Cells were washed with PBS × 1 and lysed using the ChIP kit according to the manufacturer's instructions (Abcam, Ab500). To fragment the DNA, the cells were sonicated (30 s with 30 s cooling repeatedly for 15 cycles) to obtain fragmented DNA from 100 bp to 1 kB which was verified by agarose gel migration. Lysates were subjected to immunoprecipitation overnight at 4°C with 10 μg of anti-human antibodies for GLI1 (Novus) and Histone H3 for positive control provided by the Abcam ChIP kit. The complex was subsequently incubated with Dynabeads protein G (Invitrogen) for 2 h, 4°C, on a rotating wheel. Beads were washed for 10 min at 4°C with low salt buffer (2x TE, 150 mM NaCl, 1% Triton X-100, 0.1% SDS). Beads were then washed for 10 min at 4°C with high salt buffer (2x TE, 500 mM NaCl, 1% Triton X-100, 0.1% SDS). Next, beads were washed for 10 min at 4° with LiCl buffer (1x TE, 1% NP-40, 0.25 M LiCl, 1% deoxycholate). Finally, beads were washed for 5 min at 4°C × 2 with TE Buffer (100 mM Tris- HCl, 10 mM EDTA) and eluted by heating for 10 min at 70°C with elution buffer, and incubation at 65°C overnight. Purification of DNA was accomplished using the QIAquick PCR purification kit (QIAGEN) according to the manufacturer's directions. qRT-PCR was performed to access the enrichment of the specific proteins along the NBS1 promoter using specific primer pairs: Primer-1-F: CAGTGGCCATATTATGCTACGG, Primer-1-R: GAGACCTACCACTGAGCTTC; Primer-2-F: GTCCTTGTCCAGGTCTGGCAT, and Primer-2-R: CAGGCCAAGGAGCTGAGGT.

### Plasmids and Transfection

The NBS1 cDNA plasmid was purchased from Origene. Transfection was performed using CalFectin™ Mammalian Cell Transfection Reagent (SignaGen Laboratories). The transient transfectants were harvested after 48 h for downstream processing. The stable transfectants were established by selection with puromycin for 4–6 weeks, and the surviving cells were pooled as stable mass transfectants.

### Flow Cytometry for Annexin V/Propidium Iodide (PI)

Cells were treated, in duplicate, as described in the figure legends. At the end of treatment, cells were collected by trypsinization and incubated with Annexin V FITC (BD Biosciences) and PI (Sigma) prior to analysis using a FACS Calibur flow cytometer. Data was analyzed using FlowJo software.

### GLI-Luciferase Assay

The 12 GLI-binding site-driven luciferase reporter plasmid (2 μg, GLI-luc, gift from Dr. Rune Toftgard, Karolinska Institute) and Renilla luciferase (0.2 μg, pRLTK) were co-transfected into HT29 cells using Lipofectamine 3000 (Invitrogen). Twenty four hours post-transfection, cells were exposed to GANT61 or SRI-38832 (20 μM) for 24 h, subsequently harvested using the Dual luciferase reporter assay system (Promega) according to the manufacturer's protocol. Luciferase activity was normalized to Renilla luciferase activity as a control for transfection efficiency.

### COMET Assay

Cells were treated with/without GANT61 (20 μM), SRI-38832 (20 μM) or 5-FU (20 μM) for 48 h, subsequently trypsinized and washed in PBS before being added to preheated (37°C), low-melting point agarose. The solution was pipetted onto slides pre-coated with 1% agarose. The chilled slides were allowed to lyse for 40 min at 4°C in 2.5 M NaCl,100 mM EDTA (pH 10), 10 mM Tris Base, 1% SDS, 1% Triton X−100 prior to immersion in alkaline electrophoresis solution (300 mM NaOH, 1 mM EDTA, pH 13). After 30 min, slides were placed into a horizontal electrophoresis chamber for ~30 min (1 V/cm at 4°C). The slides were washed with deionized H_2_O to remove the alkaline buffer, dehydrated in 70% ice–cold EtOH and air-dried overnight. Slides were stained with SYBR-Green and examined by microscopy. Tail length (TL) was used to quantify the DNA damage. Image analysis and quantification has been performed with ImageJ.

### Cell Proliferation Assay

Confluent monolayers of cells were trypsinized, and 10,000 cells suspended in 200 μl of medium were added to each well of the 96-well plate. After adherence, cells were treated with series of concentrations of GANT61, SRI-38832, or 5-FU (2.5–40 μM) for 72 h. The proliferation of the cells was determined using the CellTiter-Glo Luminescent Cell Viability Assay kit (Promega).

### Tumor Xenograft Model

Ten million tumor cells from culture of HT29 human colon tumor were implanted SC on the right flank into 70 NCr-nu/nu mice using a 23-gauge needle. The injection volume was 0.1 mL. The date of tumor implant recorded as Day 0. Treatment began when mice had tumors of ~75–126 mg. The dose formulation of SRI-38832 or Adrucil (5-FU) was prepared in vehicle to contain a nominal concentration of 1.0 mg/ml for IP dosing. Injection volume was 10 ml/kg, once a day for 14 days. The mice were weighed daily during the treatment interval and then twice weekly following the last injection. Tumor measurements were taken twice weekly. Length and width were measured for each tumor. Tumor volume was determined using the formula for an ellipsoid sphere: (Length × Width^2^)/2 = Volume (mm^3^). This formula was also used to calculate tumor weight, assuming unit density (1 nm^3^ = 1 mg). The experiment lasted for 60 days from the day of tumor implant. All tumors were collected at euthanasia for further analysis.

### Statistical Analysis

All statistical analysis and graphs were generated in Prism GraphPad 7.05. For linear correlation, linear regression was performed with Pearson correlation analysis. For non-parametric analysis, Mann-Whitney *u*-tests were performed. For parametric analysis of two groups, Student's *T*-test with Welch's correction were utilized. For comparing more than two groups, One-way ANOVA was performed with Tukey multiple comparisons. For comparing more than two group across a time-point study, Two-way ANOVA with Tukey multiple comparisons were performed. To identify outliers, the ROUTS test at 1% was used.

## Results

### Clinical and Prognostic Correlation Between NBS1 and GLI1 Expression in CRC Patients

**Figure 1:**
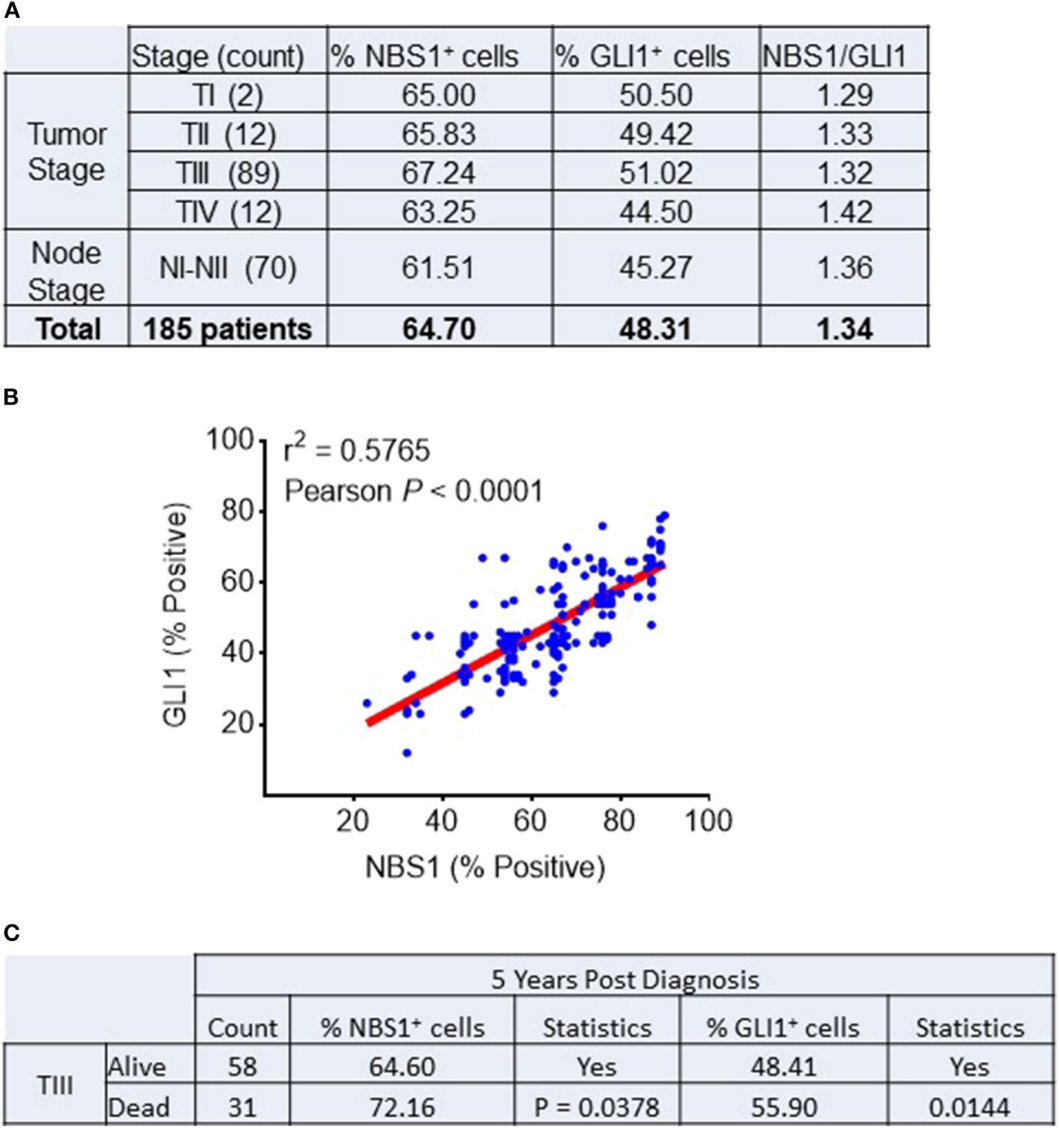
Correlation between NBS1 and Gli1 expression in colorectal cancer patients. **(A)** A cohort of 185 colorectal patient biopsies were assembled. 89.9% of patients expressed NBS1 at an average expression level of 64.7%, while 64.4% of the patients expressed GLI1 with an average expression level of 48.3%. The patients in Stage TIII had both strongest expressions of NBS1 (67.2%) and GLI1 (51.0%), while the patients in NI-II stage showed lowest expression of NBS1 (61.5%) and GLI1 (45.2%). The expression ratio of GLI1/NBS1 was about 1.34. **(B)** There was significant correlation between the two protein's expressions (*P* < 0.0001). A positive relationship between NBS1 and GLI1 protein expression levels was observed (*R*^2^ = 0.5765). **(C)** The overall survival rate of Stage III patients was 65.2% (58/89), with NBS1 and GLI1 expression (64.6 and 48.4%) at time of biopsy on lower than the average expression. In contrast, the 31 TIII Stage patients that passed away by 5 years had on average elevated NBS1 and GLI1 expression (average 72.1 and 55.9%, respectively). High expression of NBS1 and GLI1 is correlative to the poor prognosis in TIII Stage patients.

A cohort of 185 colorectal patient biopsies was assembled and staged by two clinical pathologists at First Affiliated Hospital (Xian Jiaotong University). The staging system used in this study was the American Joint Committee on Cancer (AJCC) TNM system that classifies: the extent (size) of the tumor (T), the spread to nearby lymph nodes (N) and the spread (metastasis) to distant sites. Additionally, the biopsies were stained for NBS1 and GLI1 quantification of protein expression. Based on our analysis (Figure 1A), ~90% of the patients expressed GLI1 and NBS1 concurrently. Independent of tumor stage, the expression ratio of GLI1/NBS1 was consistently in the range of 1.3–1.4. However, for patients at Stage TIII disease, the average GLI1 and NBS1 expression was found to be elevated when compared to the average patient cohort, while the patients in NI-II Stage showed lower than average expression. There was significant correlation between the two protein's expressions (*P* < 0.0001) as measured by Pearson's correlation coefficient. A linear regression analysis demonstrated a positive relationship between NBS1 and GLI1 protein expression levels as well (*R*^2^ = 0.5765) (Figure 1B). For patients receiving treatment (starting at Stage TIII), the 5 years follow-up post diagnosis showed that the survival rate of patients was less favorable when NBS1 and GLI1 expression levels were higher than average, which was 64 and 48%, respectively (Figures 1A,C). Furthermore, patients that had succumbed to disease at the 5 years post-diagnosis mark had statistically higher levels of both GLI1 and NBS1 expression (Figure 1C). These data strongly indicate that patients with elevated GLI1 and NBS1 expression had a poorer prognosis than patients with reduced protein expression when given the same treatment. Since all patients were administered a 5-FU associated chemotherapy (i.e., FOLFOX), this correlation suggests that oncogenic GLI1-mediated NBS1 expression is contributory to chemo-resistance, which results in a poor therapeutic outcome with 5-FU-associated chemotherapy treatment.

### CRC Cells With High Expression of GLI1 and NBS1 Exhibit Strong 5-FU Resistance

**Figure 2:**
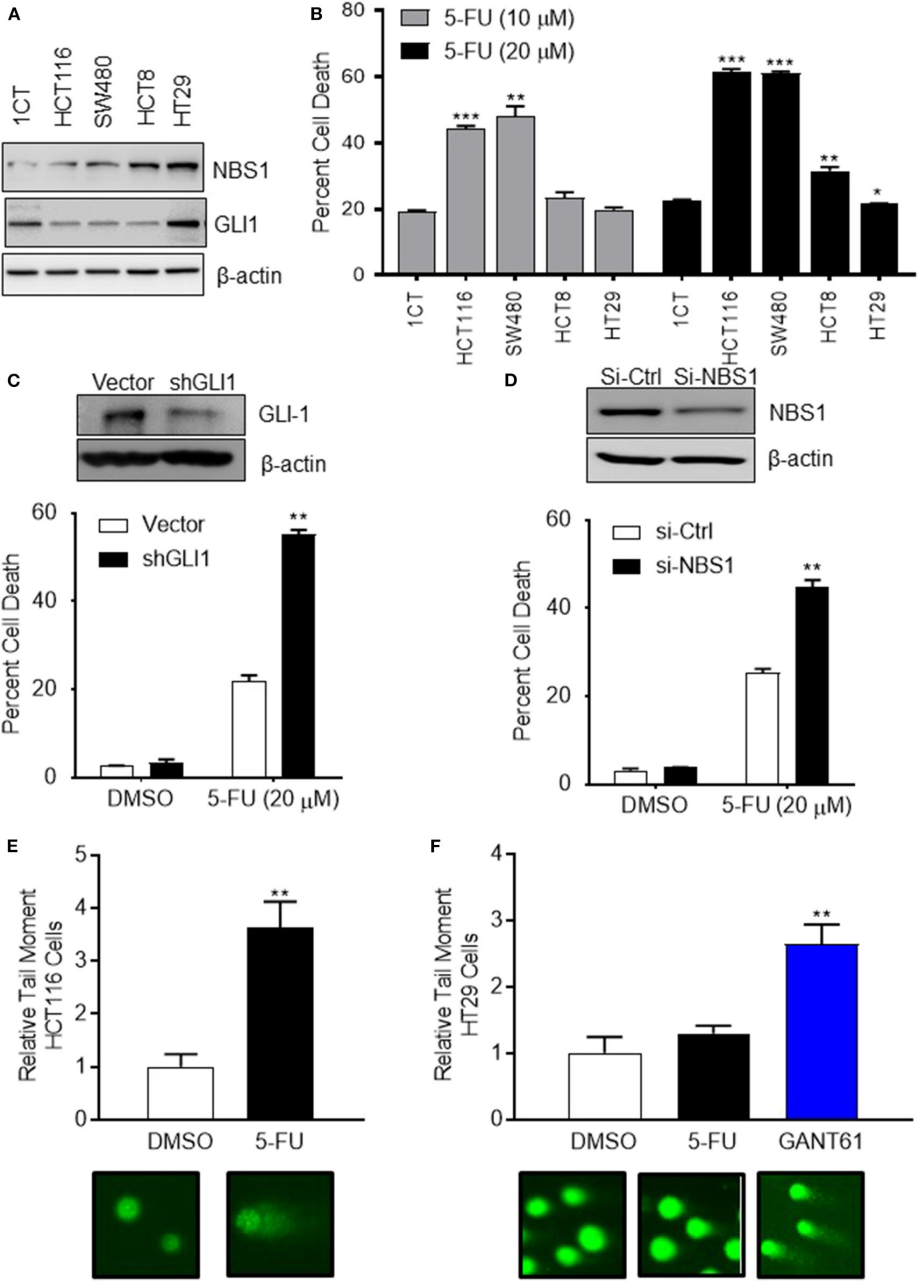
CRC cells with high expression of GLI1 and NBS1 exhibit strong 5-FU resistance. **(A)** One normal colorectal cell line (1CT) and four CRC cell lines (SW480, HCT8, HCT116, HT29) were analyzed in this study. Varying expression levels of GLI1 and NBS1 were shown in these cell lines. **(B)** The cells were treated with two concentrations (10 and 20 μM) of 5-FU for 72 h. The extent of cell death was determined by flow cytometry analysis following Annexin V/PI staining. SW480 and HCT116 cells expressing low GLI1 and NBS1 proteins were highly sensitive to 5-FU treatment. In contrast, HCT8 and HT29, which have high GLI1 and NBS1 basal expression were resistant to 5-FU. Statistical significance determined by comparing each cell line's cell death to control cells (1CT). **(C,D)** NBS1 or GLI1 depletion by siRNA or shRNA was transfected into HT29 cells. The extent of cell death after 5-FU treatment was determined by flow cytometry. Over 40% of siNBS1 cells were apoptotic, a two-fold increase over vector cells, while about 60% shGLI1 cells were apoptotic compared to only 20% dead cells of vector group. **(E,F)** Comet assay was performed on HCT116 (5-FU sensitive) and HT29 (5-FU resistant) cells with/without GANT61 (20 μM) or 5-FU (20 μM) for 48 h. Tail length (TL) was used to quantify the DNA damage. 5-FU induced DNA damage in HCT 116 cell line about 3.5 time of tail length than control, but did not in the HT29 cell line which exhibited strong 5-FU resistance. Instead, GANT61 induced DNA damage in the HT29 cell line 2.5 time of tail length than control. Statistical significance is shown as follows: *p < 0.05; **p < 0.01; ***p < 0.001.

To determine if the mechanism of resistance to 5-FU treatment in CRC is driven by GLI1-mediated NBS1 expression, one normal colorectal cell line (1CT) and four CRC cell lines (SW480, HCT8, HCT116, HT29), all with varying expression levels of GLI1 and NBS1 (Figure 2A and Supplemental Figure 1), were tested for sensitivity to two concentrations of 5-FU. The clinical data indicates that elevated co-expression of GLI1 and NBS1 is resistant to 5-FU regardless of dosing concentration, and similar results were observed with the cell lines tested. SW480 and HCT116 cells expressed low GLI1 and NBS1 protein by Western Blot analysis and demonstrated high sensitivity to 5-FU treatment. In contrast, HCT8 and HT29, which express GLI1 and have high basal NBS1 expression, were resistant to 5-FU (Figure 2B). These results support that GLI and NBS1 co-expression and function may play a critical role in 5-FU resistance.

To confirm this hypothesis, either GLI1 (Figure 2C) or NBS1 (Figure 2D) depletion by shRNA or siRNA, respectively, was performed using the 5-FU resistant line HT29. The extent of cell death after 5-FU treatment was determined by cell viability assay and apoptotic progression was determined flow cytometric analysis using Annexin V/PI staining. Statistical analysis indicated that a significant increase in apoptotic cells was observed regardless of whether cells were depleted of GLI1 or NBS1 and treated with 5-FU (Figures 2C,D). Since previous work in our lab (27) indicated that GLI1 inhibition induced DNA damage as shown γH2AX foci formation, a comet assay was performed to determine whether GLI inhibition induces apoptosis in a similar manner as 5-FU in CRC cells with GANT61 or 5-FU treatment. The assay demonstrated that 5-FU treatment induced significant DNA damage based on the results of tail length in HCT-116 cell line (5-FU sensitive) (Figure 2E), but did not in the HT29 cell line (Figure 2F), confirming the previously observed 5-FU resistance. However, treatment with GANT61, a small molecule GLI inhibitor, did induce DNA damage in the HT29 cell line (Figure 2F). These results strongly suggest that defective, and likely overactive, DNA damage repair mechanism in HT29 cells contributes to the resistance to 5-FU, and that the NBS1 overexpression could be contributory to this resistance.

### GANT61 Induces DNA Damage by Down- Regulating NBS1 Expression and Impairing MRN Complex Formation

**Figure 3:**
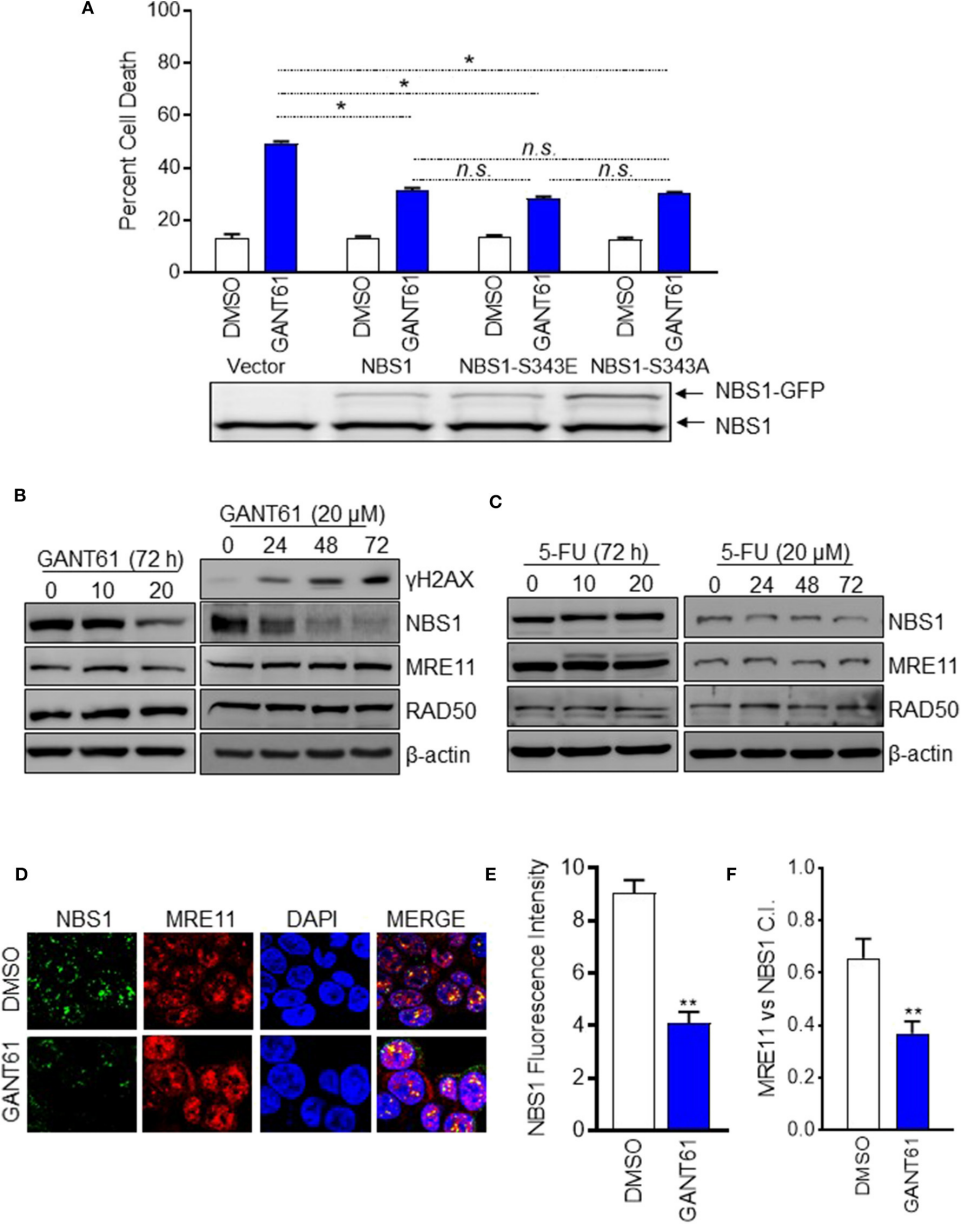
GANT61 induced cell apoptosis by down-regulation of NBS1 expression and impairing MRN complex formation. **(A)** Stable overexpression of GFP tagged NBS1 or phospho-mimic (NBS1-S343E), or NBS1 domain-negative (NB1-S343A) were treated with/without GANT61 (20 μM) for 72 h. The extent of apoptotic cell was determined by flow cytometry. NBS1 protein expression level was determined by Western-blot. Upper band represents exogenous NBS1. Lower band represents endogenous NBS1. **(B,C)** HT29 cells were treated with/without GANT61 or 5-FU at 20 μM for 72 h, and the harvested cells were subjected to western blot analysis. Increasing levels of γ-H2AX and decreasing levels of NBS1 were observed. 5-FU treatment did not result in any significant decreases of NBS1, MRE11, or RAD50 protein. **(D)** Cells treated with/without GANT61 (20 μM) for 48 h were performed by Confocal analysis. The MRN complex was examined by the co-localization of NBS1 and MRE11. **(E)** The co-localization index for specific foci and fluorescent intensity were calculated by ImageJ. **(F)** As such, a significant decrease in the co-localization of NBS1 and MRE11 was detected in GANT61-treated HT29 cells. Statistical significance is shown as follows: *p < 0.05; **p < 0.01.

In our previous publication, we reported phosphorylated NBS1 (S343) was significantly reduced after GANT61 treatment (28). We had proposed GLI1 inhibition impacts ATM-NBS1 pathway to induce cell apoptosis via hyper-phosphorylation of NBS1 (28). To test this hypothesis, we overexpressed wild type NBS1, domain-negative NBS1 (S343A), or phospho-mimic NBS1 (S343E) in HT29 cells. It was expected that the S343E mutant would be more effective in rescuing the cells while the S343A mutant would mimic GANT61 treatment. We found overexpression of any NBS1 vector rescued ~25% of cells from apoptosis after GANT61 treatment and was statistically significant. The overexpression of S343E, S343A, or total NBS1 were not statistically different from one another, indicating that total levels of NBS1, elevated by GLI1 transcription, rather than the phospohorylation status, was responsible for protection from GANT61-induced apoptosis (Figure 3A).

Since it was apparent that the NBS1 effect was not linked to a post-translational modification, we examined whether total protein levels were driving the DDR hyper-activation. This was confirmed by Western-blot data showing that significantly decreased total NBS1 levels correlated with increased levels of γ-H2AX after 72 h of GANT61 treatment (Figure 3B). Comparatively, GLI1 inhibition did not substantially alter the expression of the other components of the MRN complex, MRE11 and RAD50 (Figure 3B). Not unexpectedly, 5-FU treatment did not result in any significant decreases of NBS1, MRE11, or RAD50 protein expression under the same experimental conditions (Figure 3C). These results, together, demonstrated GLI1 inhibition reduces total NBS1 expression levels rather than ATM-mediated phosphorylation of NBS1 as we previously reported, indicating transcriptional regulation rather than post-translational modification is critical to resistance.

To investigate whether the MRN complex formation was impacted when GLI was inhibited, co-localization of NBS1 with MRE11 was examined by confocal microscopy in HT29 cells both before and after treatment with GANT61 (20 μM) for 24 h (Figure 3D). After GANT61 treatment, the fluorescent signal of NBS1 was significantly reduced (Figure 3E). As such, a subsequent decrease in the co-localization of NBS1 and MRE11 was detected in GANT61-treated HT29 cells (Figure 3F). This suggests that GLI1 activity promotes MRN complex formation through NBS1 expression, and inhibition of that activity results in failed DNA repair.

### Determination of NBS1 as a Transcriptional Target of GLI1

**Figure 4:**
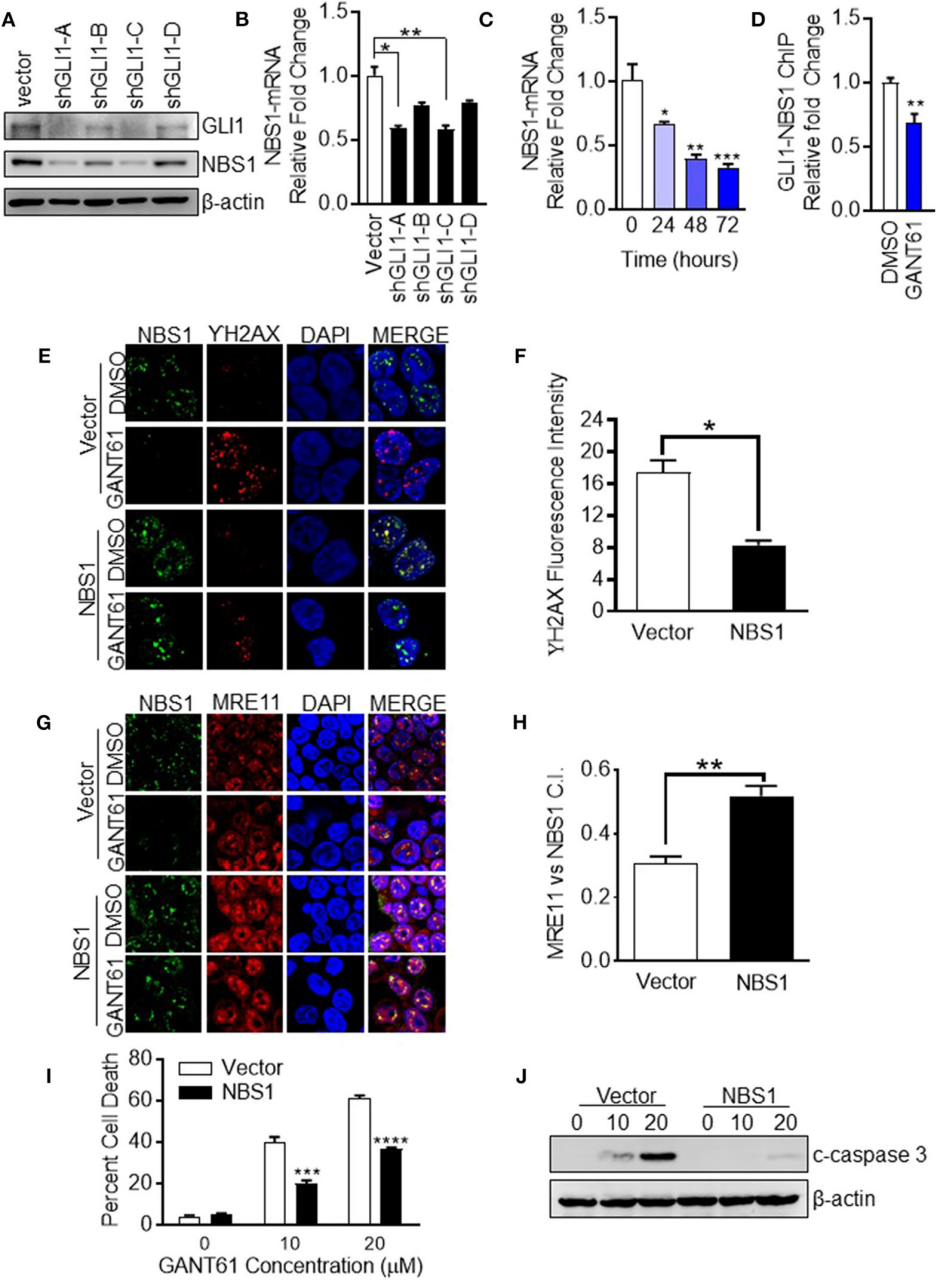
NBS1 is a transcriptional target of GLI1. **(A)** Transient knock-down GLI1 was performed in HT29 cells. The harvested cells were subjected to western blot analysis. **(B,C)** NBS1 mRNA expression was determined by qRT-PCR in each shGLI1 transfectants or cells with GANT61 treatment. NBS1 mRNA was significantly decreased in shGLI1 cells or GANT61 treated cells. **(D)** Analysis of the promoter sequences of NBS1 revealed a putative GLI binding sequences in the promoter: AACCCCCCA (site 1, promoter). The pair of specific primes were designed and generated to detect potential binding enrichment (Supplemental Figure 1). HT29 cells were treated with/without GANT61 (20 μM) for 24 h. Then, cells were harvest to perform ChIP assay which was performed by employing GLI1 immunoprecipitation and DNA sequencing using primers that flanked the putative GLI binding sequence. The enrichment was assayed by qRT-PCR method. **(E–H)** Transient overexpression of NBS1 cells were performed to confocal analysis to detect NBS1 and γ-H2AX or NBS1 and MRE11 colocaliztion. As such, a decreased γ-H2AX intensity and significant recovery in the co-localization of NBS1 and MRE11 was detected in NBS1 transfected cells with GANT61 treatment. **(I,J)** Overexpression NBS1 also reduces GANT61 induced apoptosis. Statistical significance is shown as follows: *p < 0.05; **p < 0.01; ***p < 0.001; ****p < 0.0001.

The determination of NBS1 as a GLI1 transcriptional target required biochemical analysis at multiple levels. First, to confirm down-regulation of NBS1 at both the transcriptional and translational levels was caused by GLI1 inhibition, transient knock-down of GLI1 using shRNA in HT29 cells, which resulted in significantly reduced the NBS1 protein (Figure 4A) and mRNA level (Figure 4B). Furthemore, qRT-PCR analysis indicated that upon GANT61 treatment, the NBS1 mRNA significantly decreased as early as 24 h post treatment (Figure 4C), implicating GLI1 involvement in the regulation of NBS1 transcription. To determine whether GLI1 directly regulates NBS1 transcription, the promoter sequences of NBS1 was analyzed revealed a putative GLI binding site (Supplemental Figure 2). Critical to the hypothesis that GLI1 is the transcriptional regulator of NBS1, we investigated whether GLI1 binds to the NBS1 promoter via a chromatin immunoprecipitation (ChIP) assay. GLI1 binding enrichment was observed and this binding was significantly reduced when GLI was inhibited by GANT61 after 24 h (Figure 4D). These results indicate that NBS1 is, in fact, a novel transcriptional target of GLI1.

### Overexpression of NBS1 Alleviates GLI1 Inhibition Induced DNA Damage and Cell Apoptosis

To determine whether NBS1 was a critical effector of GLI1 inhibition, NBS1 was transiently overexpressed in HT29 cells for 48 h prior to treatment with GANT61 (20 μM) for 24 h. The fluorescent signal of γ-H2AX was drastically reduced when NBS1 was overexpressed compared to the vector transfected cell line (Figures 4E,F). As such, a significant recovery in the co-localization of NBS1 and MRE11 was detected in NBS1 transfected cells with GANT61 treatment (Figures 4G,H). Moreover, percent of cell death was significantly reduced (Figure 4I) and caspase 3 cleavage was not detectable in NBS1 overexpressed cells (Figure 4J), indicating cells were rescued from GANT61 induced apoptosis.

### GLI1 as a Potential Therapeutic Target in BRAF^V600E^ Mutant Cells

**Figure 5:**
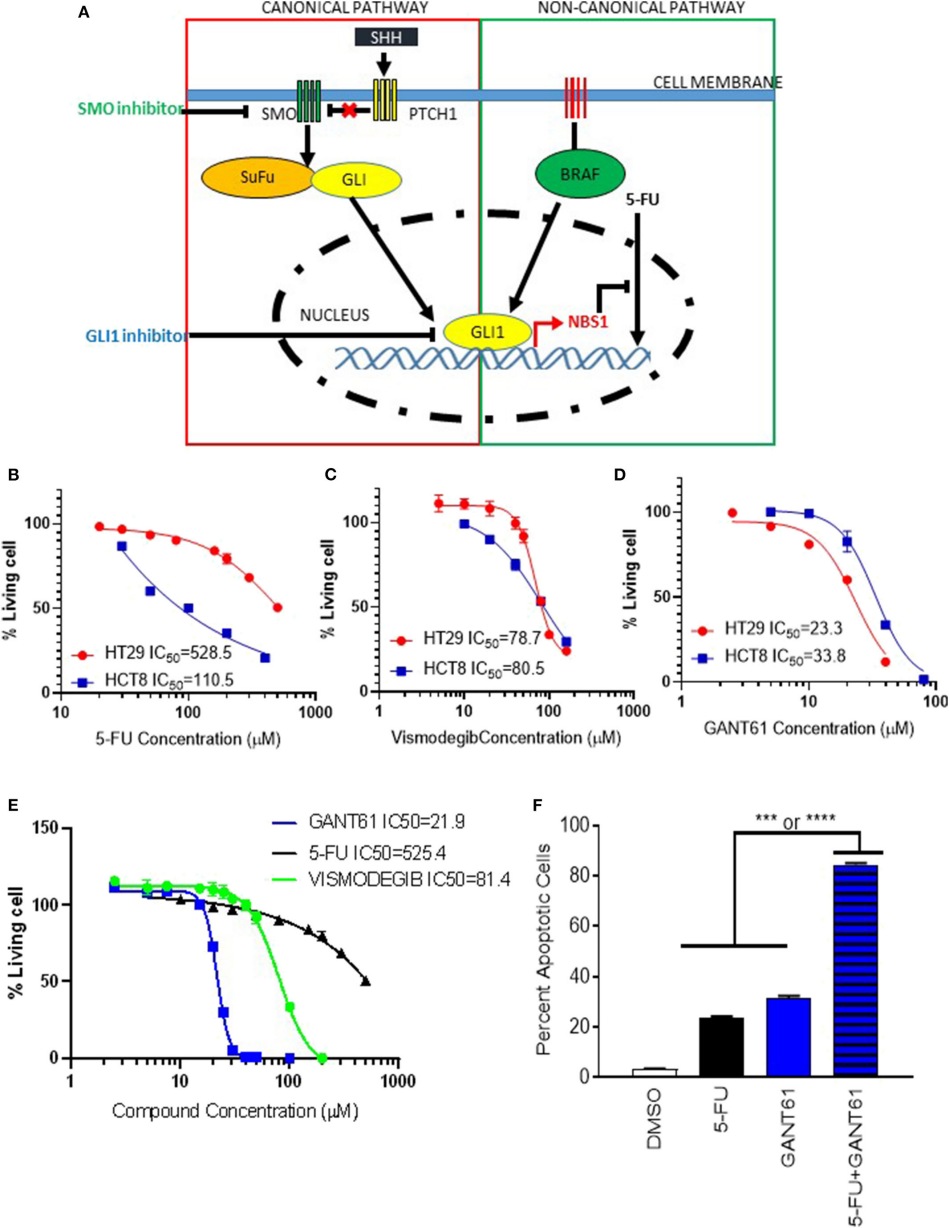
GLI1 as a potential therapeutic target in BRAF^V600E^ mutant cells. **(A)** The scheme of GLI1 regulating NBS1 expression induced 5-FU resistance. While SMO inhibitors block classical (Hh ligand-dependent) signaling, they are unable to block non-classical canonical oncogenic GLI1 activation pathways, like KRAS/B-RAF. By targeting GLI1 at the juncture of the hedgehog pathway, which has oncogenic activity, and the KRAS/B-RAF pathway, the parallel oncogenic signals can both be mitigated. **(B–D)** the HCT8 (low GLI1 expression) and HT29 (high GLI1 expression) cell lines were treated with 5-FU, GNAT61, and VISMODEGIB, respectively to assay the GLI1 target therapeutic effect. **(E)** A comparison of VISMODEGIB or GANT61 or 5-FU effect on HT29. Data was determined using the CellTiter-Glo Luminescent Cell Viability Assay kit. IC50 was calculated by Graphpad Prism 8. **(F)** Combination of GANT61 and 5-FU exhibits synergistic effect. HT29 cells were treated with 10 μM of 5-FU or GANT61 or combined 5-FU and GANT61 for 72 h. The extent of cell death was determined by flow cytometry analysis following Annexin V/PI staining. Statistical significance is shown as follows: ***p < 0.001; ****p < 0.0001.

Since GLI1 resides at the intersection of canonical (Hedgehog) and non-canonical pathways (BRAF), a GLI1 specific inhibitor would be more effective than canonical pathway inhibitors (Figure 5A). In order to investigate whether GLI1 is a targeted therapeutic strategy in BRAF variants, HCT8^BRAFWT^ (low GLI1 expression) and HT29^BRAFV600E^ (high GLI1 expression) were analyzed (Figure 2A and Supplemental Figure 1). Lower GLI1 expression lead to increased sensitivity to standard-of-care 5-FU treatment in HCT8 cells, with an IC_50_ 5-fold lower than HT29 cells (Figure 5B). HCT8 and HT29 cells were treated with Vismodegib (a Hh pathway SMO inhibitor) and GANT61 to address canonical and non-canonical GLI activation. Vismodegib had the same IC_50_ on both HT29 and HCT8 cell lines, indicating cell death induced by canonical hedgehog/SMO inhibition is non-specific since elevated GLI1 levels in HT29 are driven by non-canonical activation (Figure 5C). In contrast, the IC_50_ of GANT61 is increased in HCT8 which is expected since these cells are GLI1-independent (Figure 5D). Furthermore, the IC_50_ of Vismodegib is ~80 μM, 4-fold higher than GANT61 in HT29 cells (Figure 5E), indicating that the Hh pathway is not the dominant driver of oncogenic signaling in these cells. Therefore, by directly targeting the effector protein GLI1, we have identified a novel therapeutic target in BRAF variants with elevated GLI1 expression.

### Pharmacological Inhibition of GLI Alleviates 5-FU Resistance

To determine if pharmacologic inhibition of GLI1 could provide as a combinatorial therapy approach, HT29 cells were treated with a combination of 5-FU and GANT61. Although individual treatment with GANT61 resulted in an IC_50_ of 21.9 μM while 5-FU required 625.4 μM, using half the IC_50_ of GANT61 (10 μM) and a suboptimal dose of 5-FU (20 μM), we observed a synergistic effect with statistical significance when compared to either treatment on its own (Figure 5F). Therefore, we believe a novel application for GLI1-inhibitors is combination therapy with 5-FU to reduce chemo-resistance in CRC treatment.

### Identification of a Viable Alternative to GANT61 for Therapeutic Targeting of GLI1

**Figure 6:**
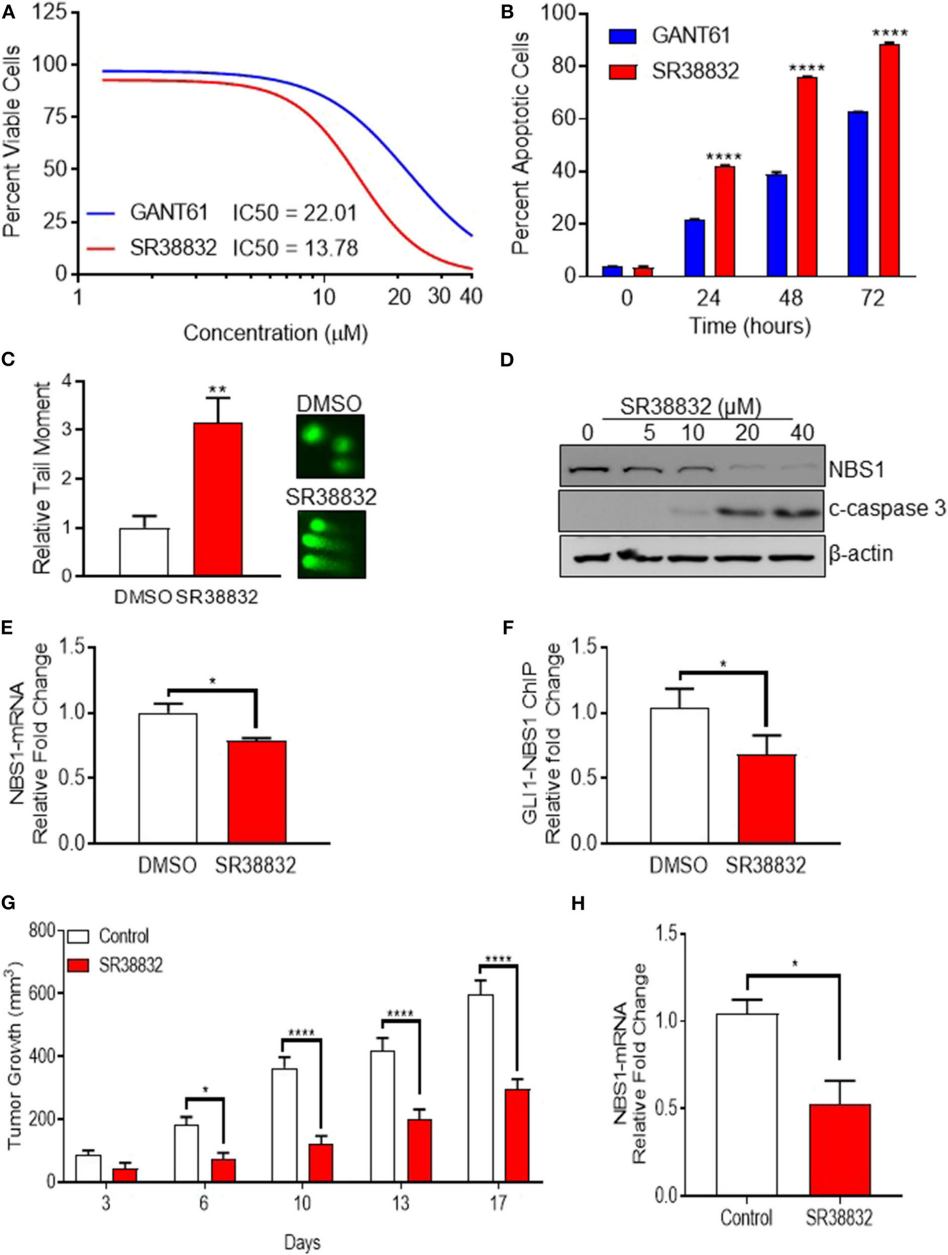
Identification of a viable alternative to GANT61 for therapeutic targeting of GLI1. **(A,B)** The novel GLI1 inhibitor, SR38832, exhibits more effective anti-cancer activity than GANT61. **(C–E)** SR38832 induces DNA damage through the same mechanism as GANT61 to down-regulate NBS1 expression. **(F)** The harvested cells were also subject to ChIP assay as the same protocol as mentioned before. NBS1 mRNA levels were decreased with SR38882 treatment. **(G,H)** SR38832 significantly exhibits the anti-tumor effect in HT29 xenograft model. Ten million tumor cells from culture of HT29 human colon tumor were implanted SC on the right flank into 70 NCr-nu/nu mice. Treatment began when mice had tumors of ~75–126 mg. The dose formulation of SR38832 was prepared in vehicle to contain a nominal concentration of 1.0 mg/ml for IP dosing. Injection volume was 10 ml/kg, once a day for 14 days. **(G)** A robust decrease of tumor weight was observed in SR38832 group. **(H)** When the experiment was terminated, tumor sample was taken out and mashed into single cells. mRNA was extracted, qRT-PCR was performed to assay NBS1 mRNA level. Statistical significance is shown as follows: *p < 0.05; **p < 0.01; ****p < 0.0001.

While GANT61 is useful for *in vitro* pharmacological inhibition assays, it becomes hydrolyzed and loses biological activity *in vivo* (29). Indeed, an analysis of the absorption, distribution, metabolism, and excretion (ADME) properties determined that solubility and stability values could not be determined (Supplemental Table 1A). Additionally, and equally problematic, GANT61 acts as a pan-inhibitor of GLI family proteins, and the GLI-NBS1 phenomenon is specific to GLI1 activity. Therefore, in order to identify targeted GLI1 inhibitors, a small panel of proprietary compounds with similar chemical properties to GANT61 were identified and screened for GLI inhibition. For the primary screen, HT29 cells were transfected with a GLI1 luciferase reporter plasmid, then treated with the selected compounds (Supplemental Figure 3A). A secondary assay for apoptotic activity was used to remove any false positives (Supplemental Figure 3B). Compounds from these screens were selected if they met a threshold anti-GLI1 function on par with GANT61, reducing the luciferase signal to a level <50% of the level observed in a DMSO-treated control, as well as induced cell death. From these two assays, SRI-38832 was identified as the lead compound, as it showed the greatest GLI1 inhibition and promoted the most cell death. Dose response using the CellTiterGlo assay determined the IC_50_ of SRI-38832 was 13 μM, lower than that of GANT61 (21 μM) (Figure 6A). A time-course assay showed that as early as 48 h of treatment with SRI-38832, ~80% of HT29 cells were apoptotic, statistically greater than the nearly 40% seen in GANT61 treated cells (Figure 6B). 1CT (normal colon epithelia cell) or HT29 cell lines were used to ensure SRI-38832 was not cytotoxic (Supplemental Figures 3C,D).

To ascertain whether SRI-38832 has the same mechanism of activity as GANT61 (i.e., cell death by NBS1 downregulation), comet assays and NBS1 regulation assays were performed. The comet assay results demonstrated SRI-38832 induced DNA damage in the same manner as GANT61 (Figure 6C). A significant decrease in NBS1 protein and increased production of cleaved caspase-3 in a dose-dependent manner was observed with SRI-38832 treatment (Figure 6D). NBS1 mRNA levels were decreased with SRI-38882 treatment (Figure 6E) and a ChIP assay showed that SRI-38832 treatment resulted in a significant decrease of enrichment of GLI1 on the NBS1 promoter (Figure 6F). Based on the confirmation of the mechanism of action, a preliminary pharmacokinetic assessment of SRI-38832 found that based on half-life, plasma-retention, and stability, this compound was suitable for proof-of-concept *in vivo* testing (Supplemental Tables 1B,C).

### SRI-38832 Significantly Reduces Tumor Burden in an HT29 (5-FU Resistant) Xenograft Model via a GLI1-Dependent Mechanism

Based on the aforementioned *in vitro* results, we next evaluated the *in vivo* therapeutic potential of GLI1-inhibition with SRI-38832 on the growth of HT29 xenografts in nude mice. A robust decrease of tumor weight was observed in SRI-38832 treated mice compared to vehicle injected control mice. A reduction in tumor growth was observed as early as 3 days post-treatment start date and after 7 days post-treatment start date, tumor mass in the SRI-38832 group reduced by >50% of the control group. Body weight of the animals was monitored during the experiment, and no significant loss of body weight was observed in SRI-38832 treated group (data not shown). At the point of termination, the size and mass of tumors from the SRI-38832 group were statistically less than control (>60% difference) (Figure 6G). Tumor samples were evaluated for NBS1 expression by qPCR to determine whether the mechanism of action *in vivo* was consistent with *in vitro* observations. As shown in Figure 6H, NBS1 mRNA expression levels in the SRI-38832 treated group were significantly decreased relative to control. These findings mirrored the *in vitro* findings, showing that SRI-38832 inhibits DDR in tumors by decreasing NBS1 levels, resulting in diminished tumor size and improved outcome *in vivo*.

## Discussion

BRAF mutations are often associated with aggressiveness, poor differentiation and resistance to therapy in CRC (2). CRC patients with BRAF V600E mutations exhibit poor prognosis and response to conventional cytotoxic chemotherapy (FOLFOX)/(FOLFIRI) or novel target chemotherapy, such as panitumumab and cetuximab, monoclonal antibodies targeting the epidermal growth factor receptor (EGFR/ERBB1) (30). Given the aggressive nature of *BRAF*^*V*600*E*^-driven disease, clinicians are often faced with the challenge of achieving initial disease control in patients at risk for rapid clinical deterioration.

Studies to-date have typically focused on targeting GLI inhibition through the canonical Hh pathway, targeting upstream regulators like SMO, and subsequently sequestering GLI1 in the cytoplasm. Variable success using SMO inhibitors has been demonstrated in preclinical models (31–37) and clinically (38–43), in a variety of different types of human cancers. This is due to the predominant dependence of certain types of human cancers on canonical Hh signaling, such as basal cell carcinoma (39, 43) and medulloblastoma (38). However, for CRC trials, SMO inhibitors universally have not provided any substantial clinical benefit (44, 45). This is likely because over 50% of colorectal and pancreatic cancers bear KRAS or BRAF mutations, which can result in a non-canonical constitutive activation of GLI1 (46–48).

Currently, the overall response rate to 5-FU and its combinational treatment (FOLFOX) is limited to 10–15% due to inherent or acquired chemo-resistance in the clinic. Despite this, 5-FU-based chemotherapy is still the first line treatment option for advanced CRC patients (49, 50). According to the clinical 5 years follow up data presented herein, we first reported that patients with high GLI1 and NBS1 expression consistently have poor treatment outcomes. Since all patients in this study received 5-FU related chemotherapy, the GLI1-NBS1 axis emerged as a putative prognostic indicator of chemotherapeutic effectiveness with standard-of-care therapies (i.e., FOLFOX). *In vitro*, we found cells with high GLI1 and NBS1 expression exhibited strong 5-FU resistance. Knocking-down of GLI1 or NBS1 expression rendered inherently resistant cell lines sensitive to 5-FU treatment. Subsequent identification of a GLI1 target sequence in the NBS1 promotor region tethered oncogenic GLI1 transcriptional activity to elevated NBS1 expression. Therefore, we demonstrate that oncogenic upregulation of GLI1 amplifies NBS1 expression, resulting in an enhanced DDR in a subset of cancer cells, in turn promoting survival in high genotoxic stress environments. Additionally, we show that this mechanism is a critical driver of chemo-resistance in the presence of DNA damaging agents.

In order to reduce DDR-related chemo-resistance, several strategies utilizing chemotherapeutic combination therapy are in clinical trials. The core concept of these trials is to limit the DDR mechanism when DNA damage is induced; however, the challenge is to specifically alleviate DDR in cancer cells without affecting the normal and necessary functions of DDR. Ataxia-telangiectasia mutated (ATM) is a kinase that regulates a number of substrates including the phosphorylation of NBS1 to initiate and enhance its DDR activity. As such, many programs have attempted to develop various ATM inhibitors aimed to inhibit DDR (51). However, ATM itself is not a specific therapeutic target because of its multiple dominant nature, critical kinase function in normal cellular processes, and essential role in the maintenance of chromosome integrity at all phases of the cell cycle (52). In this study, diminished DDR is achieved through down-regulation of NBS1 expression via GLI1 inhibition, resulting in the failure to form the MRN complex and induce apoptosis. Since GLI1 is not typically expressed by differentiated cells, targeting oncogenic expression of GLI1 would result in fewer off-target effects and provide a specific therapeutic strategy.

Altogether, we demonstrate GLI1 inhibitors provide a promising chemotherapy approach in either single dose as a target therapy against BRAF mutant CRCs or as a DDR reducer to use in combination with conventional DNA damage inducing chemotherapeutics. However, a significant therapeutic barrier is lack of viable *in vivo* GLI1 inhibitor. GANT61, the most commonly used pan-GLI family inhibitor, has only been shown to be effective when administered by subcutaneous injection due to its unstable structure and poor PK file (29). In order to circumvent this barrier to *in vivo* evaluation, a novel GLI1 antagonist generated at Southern Research, SRI-38832, is reported. Like GANT61, SRI-38832 can specifically down-regulate GLI1-NBS1 mediated transcription by preventing GLI1 binding to the NBS1 promoter. This novel GLI1 inhibitor exhibits a lower IC_50_ than that of GANT61 and, most importantly, has stable pharmacokinetic/bioavailability profiles and is effective in xenograft murine model, making it a novel scaffold for hit-to-lead optimization. The identification and development of SRI-38832 is a critical first step toward a clinically viable compound, as it is a first-in-class GLI1 inhibitor to show efficacy by i.p., injection *in vivo*. Future medicinal chemistry efforts will be focused on increasing the potency, improving the global pharmacokinetic properties and optimizing the structure to improve stability and increase specificity, providing a treatment option for patients with metastatic *BRAF*^*V*600*E*^ mutant CRC.

In conclusion, a novel, causal link between GLI1 activity and NBS1 transcription levels is described, where patients with high expression of these proteins were associated with poor prognosis, likely linked to chemotherapeutic resistance. Through a series of mechanistic studies, GLI1 was identified as a novel transcriptional regulator of NBS1. GLI1 inhibition by pharmacologic inhibition *in vitro* could overcome 5-FU resistance by silencing NBS1 production and inhibiting DDR. Furthermore, GLI1 specific-inhibitors are effective in CRC with non-canonical oncogenic BRAF-driven GLI expression. Finally, a novel, direct GLI1-inhibitor (SRI-38832) is presented, making it a first- in-class tool for analyzing GLI1 inhibition *in vivo*.

## Data Availability Statement

The datasets used and/or analyzed during the current study are available from the corresponding author on reasonable request.

## Ethics Statement

Patient data was a retrospective analysis of banked sample from de-identified patients at First Affiliated Hospital in Xian Jiaotong University. This data falls under Exemption 4 of 45 CFR 46 in the NIH guidelines for human subject research. All animal studies were approved by Southern Research's Animal Care and Use Program (ACUP). Animal care was in compliance with the standard operating procedures (SOPs) of Southern Research, the *Guide for the Care and Use of Laboratory Animals: Eighth Edition*, and the US Department of Agriculture through the Animal Welfare Act.

## Author Contributions

RZ: conceptualization, investigation, formal analysis, visualization, methodology, roles/writing—original draft, writing—review, and editing. JM: investigation and formal analysis. JA: data curation, investigation, formal analysis, visualization, writing—review, and editing. VS: investigation and methodology. TN: investigation, methodology, and resources. BX: data curation and resources. MS: conceptualization, funding acquisition, project administration, and supervision. RB: conceptualization, funding acquisition, investigation, project administration, supervision, writing—review, and editing.

### Conflict of Interest

The authors declare that the research was conducted in the absence of any commercial or financial relationships that could be construed as a potential conflict of interest.

## Supplementary Material

Supplemental Figure 1 HT29 express greater levels of NBS1 and GLI1 than other cell lines. Western blot bands from Figure 2A were quantified and shown as a bar graph (normalized to β-actin).

Supplemental Figure 2 Constitutive GLI1 binding sites in the NBS1 promoter. The reported constitutive GLI1 binding site is GACCACCCA. The potential constitutive GLI1 binding site was identified in the NBS1 promoter, two mismatched nucleotides indicated in red. The forward (red) or reverse (green) primers were designed to flank the putative GLI binding sequence. The length of detected fragment including binding site is 200 bp.

Supplemental Figure 3 A novel GLI1 inhibitor (SR38832) was identified. **(A)** The 12 GLI-binding site-driven luciferase reporter (2 μg) and Renilla luciferase (0.2 μg) were co-transfected into HT29 cells, After 24 h, cells were exposed to 20 μM of GANT61 or other compounds for 24 h, subsequently harvested using the Dual luciferase reporter assay system. Luciferase activity was normalized to Renilla luciferase activity. Compound was selected if it met a threshold anti-GLI1 function on par with GANT61, reducing the luciferase signal to a level <50% of the level observed in a DMSO-treated control. **(B)** HT29 cells were treated with 20 μM of GANT61 or other compounds for 72 h. The extent of cell death was determined by flow cytometry analysis following Annexin V/PI staining. **(C,D)** Cytotoxicity assay. HT-29 (colorectal cancer cell) or 1CT (normal colon cell) was treated with series of concentrations of GANT61 or SR38832 (2.5–40 μM) for 72 h. The proliferation of the cells was determined using the CellTiter-Glo Luminescent Cell Viability Assay kit.

Supplemental Table 1 **(A)** ADME profile in GANT61 and SR38832. HLM, Human Liver Microsomes; MLM, Mouse Liver Microsomes; **(B,C)** Pharmacokinetic profile. A rapid, sensitive, and selective high-performance liquid chromatography tandem mass spectrometric method (HPLC-MS) has been developed and validated for the simultaneous determination of Pharmacokinetics of SR38832 in three individual CD1 mouse plasma after I.P. administration (10 mg/kg). **(B)** Raw data. **(C)** PK Values. T1/2, The time required for the concentration of the drug to reach half of its original value; Cmax, The peak plasma concentration of a drug after administration; AUClast, Area under the curve of the integral of the concentration-time curve.

## Abbreviations

ADME: Absorption, Distribution, Metabolism, and Excretion
ATM: Ataxia-telangiectasia mutated
ChIP: Chromatin Immunoprecipitation
CRC: Colorectal Cancer
DDR: DNA Damage Repair
DSB: Double-Strand Break
EGFR: Epidermal Growth Factor Receptor
FOLFOX: FOL – Folinic acid, F – Fluorouracil (5-FU), OX – Oxaliplatin
FOLFIRI: FOL – Folinic acid, F – Fluorouracil (5-FU), IRI – irinotecan
Hh pathway: Hedgehog pathway
MRN: MRE11, Rad50, and NBS1 complex
NBS1: Nijmegen Breakage Syndrome-1
PI: Propidium Iodide
PVDF: Polyvinylidene Difluoride
SMO: Smoothened
SNP: Single-Nucleotide Polymorphisms
5-FU: Flurouracil.
