# Supplementary figures and images for: GLI1 Inhibitor SRI-38832 Attenuates Chemotherapeutic Resistance by Downregulating NBS1 Transcription in BRAFV600E Colorectal Cancer
Source: Front Oncol. 2020 Feb 28;10:241. doi: 10.3389/fonc.2020.00241 (PMC7058788; doi:10.3389/fonc.2020.00241)

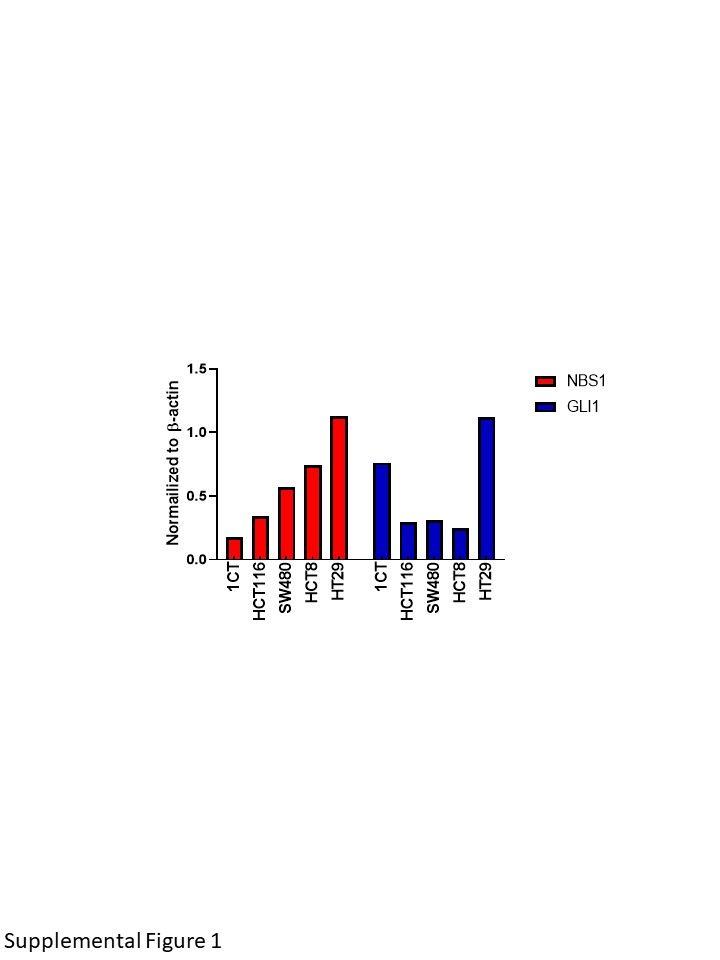

Supplement: Supplemental Figure 1 — HT29 express greater levels of NBS1 and GLI1 than other cell lines. Western blot bands from Figure 2A were quantified and shown as a bar graph (normalized to β-actin). [file Image_1.JPEG]

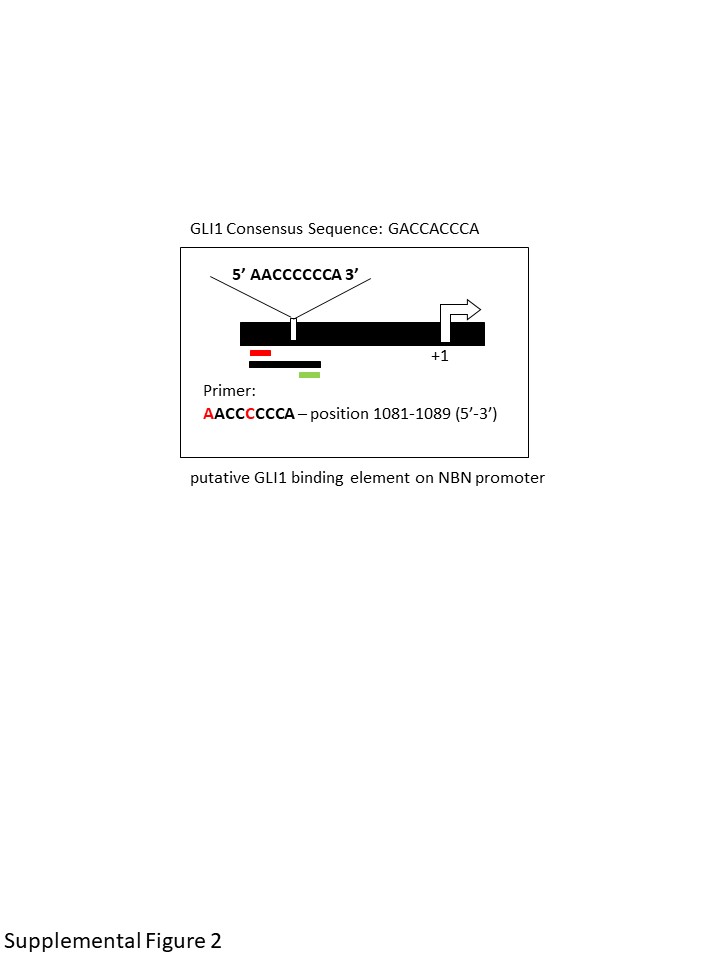

Supplement: Supplemental Figure 2 — Constitutive GLI1 binding sites in the NBS1 promoter. The reported constitutive GLI1 binding site is GACCACCCA. The potential constitutive GLI1 binding site was identified in the NBS1 promoter, two mismatched nucleotides indicated in red. The forward (red) or reverse (green) primers were designed to flank the putative GLI binding sequence. The length of detected fragment including binding site is 200 bp. [file Image_2.JPEG]

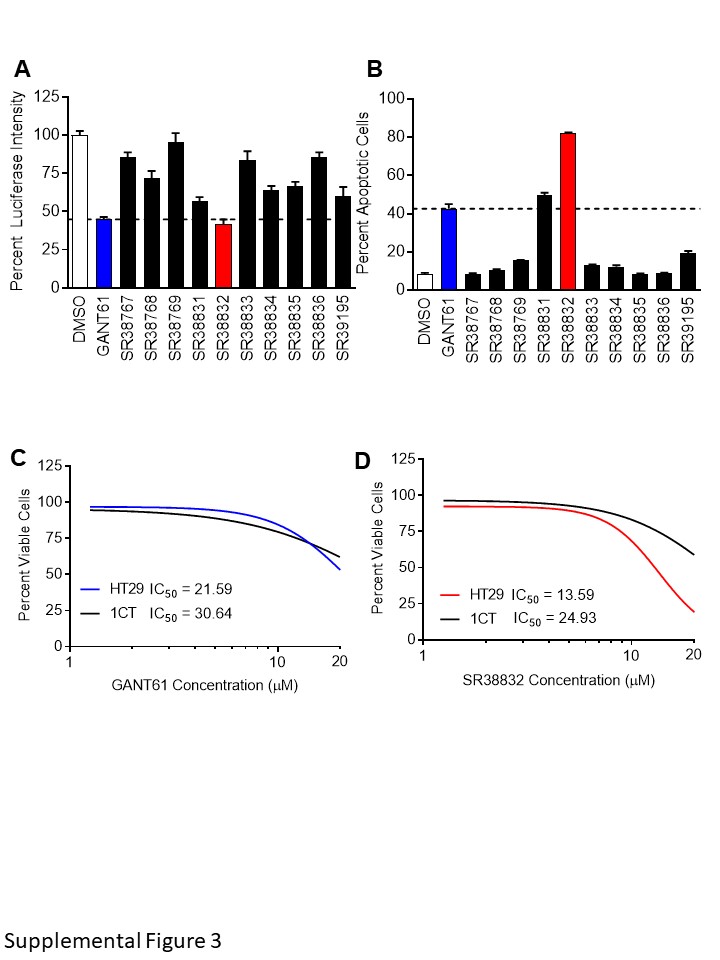

Supplement: Supplemental Figure 3 — A novel GLI1 inhibitor (SR38832) was identified. (A) The 12 GLI-binding site-driven luciferase reporter (2 μg) and Renilla luciferase (0.2 μg) were co-transfected into HT29 cells, After 24 h, cells were exposed to 20 μM of GANT61 or other compounds for 24 h, subsequently harvested using the Dual luciferase reporter assay system. Luciferase activity was normalized to Renilla luciferase activity. Compound was selected if it met a threshold anti-GLI1 function on par with GANT61, reducing the luciferase signal to a level <50% of the level observed in a DMSO-treated control. (B) HT29 cells were treated with 20 μM of GANT61 or other compounds for 72 h. The extent of cell death was determined by flow cytometry analysis following Annexin V/PI staining. (C,D) Cytotoxicity assay. HT-29 (colorectal cancer cell) or 1CT (normal colon cell) was treated with series of concentrations of GANT61 or SR38832 (2.5–40 μM) for 72 h. The proliferation of the cells was determined using the CellTiter-Glo Luminescent Cell Viability Assay kit. [file Image_3.JPEG]
