# Supplementary material for: GLI1 Inhibitor SRI-38832 Attenuates Chemotherapeutic Resistance by Downregulating NBS1 Transcription in BRAFV600E Colorectal Cancer
Source: Front Oncol. 2020 Feb 28;10:241. doi: 10.3389/fonc.2020.00241 (PMC7058788; doi:10.3389/fonc.2020.00241)
Supplement: Supplemental Table 1 — (A) ADME profile in GANT61 and SR38832. HLM, Human Liver Microsomes; MLM, Mouse Liver Microsomes; (B,C) Pharmacokinetic profile. A rapid, sensitive, and selective high-performance liquid chromatography tandem mass spectrometric method (HPLC-MS) has been developed and validated for the simultaneous determination of Pharmacokinetics of SR38832 in three individual CD1 mouse plasma after I.P. administration (10 mg/kg). (B) Raw data. (C) PK Values. T1/2, The time required for the concentration of the drug to reach half of its original value; Cmax, The peak plasma concentration of a drug after administration; AUClast, Area under the curve of the integral of the concentration-time curve. [file Table_1.pdf]

A

	GANT61	SRI-38832
Molecular Weight	429.61	404
EC_{50} (µM)	20	13.6
Solubility (µM)	UNDETECTABLE	84
Log D	UNDETECTABLE	2.5
$t_{1/2}$ (min)	UNDETECTABLE	HLM = 9.1
		MLM = 9.5

B

	Time (hours)							
	0.0833	0.25	0.5	1	2	4	8	24
Mouse 1	1209.85	772.525	697.071	513.842	318.975	54.44	8.002	BLOQ
Mouse 2	1224.916	935.732	770.726	629.448	370.906	96.035	11.699	BLOQ
Mouse 3	1186.685	1050.637	777.957	610.409	322.526	76.659	4.529	BLOQ
**Mean (ng/mL)**	**1207.150**	**919.631**	**748.585**	**584.566**	**337.469**	**75.711**	**8.077**	**NA**
SD (ng/mL)	*19.258*	*139.753*	*44.758*	*61.984*	*29.012*	*20.814*	*3.586*	*NA*

Dose = 10 mg/kg i.p.

C

	$T_{1/2}$ (hours)	C_{max} (ng/mL)	AUC_{last} (h*ng/mL)
Mouse 1	1.165	1210	1616.880
Mouse 2	1.218	1225	1976.916
Mouse 3	0.975	1187	1840.261
**Mean**	**1.120**	**1207.333**	**1811.352**
SD	*0.128*	*19.140*	*181.751*

Supplemental Tables
